# Age-related changes in central corneal thickness and their association with ocular biometric parameters in children

**DOI:** 10.3389/fmed.2026.1824418

**Published:** 2026-05-22

**Authors:** Qing Yuan, Yawen Bai, Zhe Su, Ying Zou, Yan Lu

**Affiliations:** Department of Ophthalmology, Beijing Shijitan Hospital, Capital Medical University, Beijing, China

**Keywords:** central corneal thickness, corneal curvature, myopia, ocular biometry, pediatric ophthalmology

## Abstract

**Objective:**

To investigate age-related changes in central corneal thickness (CCT) and its relationship with ocular biometric parameters in children.

**Methods:**

This retrospective study included 393 children (786 eyes) examined using ZEISS IOLMaster 700 to measure CCT, axial length (AL), corneal curvature (Km), anterior chamber depth (ACD), and lens thickness (LT). Associations between CCT and age, sex, refractive status, and these biometric parameters were analyzed. Longitudinal CCT changes over 1 year were assessed in 158 children.

**Results:**

The cohort comprised 192 males (48.8%) and 201 females (51.2%) with a mean age of 9.5 ± 3.1 years and mean CCT of 551.53 ± 32.93 μm. CCT showed weak but significant positive correlations with age (*r* = 0.150, *p* < 0.001), AL (*r* = 0.121, *p* = 0.001), and ACD (*r* = 0.079, *p* = 0.026), and a negative correlation with Km (*r* = −0.239, *p* < 0.001). No significant correlation was found with sex or LT. Myopic eyes had thinner CCT than non-myopic eyes (548.77 ± 34.37 μm vs. 554.20 ± 31.29 μm, *p* = 0.021), and spherical equivalent was negatively correlated with CCT (*r* = −0.163, *p* = 0.003). Longitudinally, CCT increased significantly over 1 year in children aged 3–12 years, with no significant change in those aged 13–17.

**Conclusion:**

CCT increases with age in children aged 3–12 years and stabilizes after 13 years. Thinner CCT is associated with myopia and steeper corneal curvature, informing pediatric eye assessment, myopia management, and orthokeratology safety.

## 1 Introduction

Central corneal thickness (CCT) refers to the distance between the anterior surface of the corneal epithelium and the posterior surface of the endothelium in the central region of the cornea (1). As a key ocular biometric parameter, CCT plays a pivotal role in the diagnosis, management, and prognosis of numerous ophthalmic conditions. It serves as a vital indicator for evaluating corneal morphology and is essential in both pediatric and adult ophthalmic assessments. Notably, a thinner CCT has been identified as a risk factor for primary open-angle glaucoma (2, 3). In the context of orthokeratology lens fitting, CCT is a critical safety measure, as excessively thin corneas may lack sufficient biomechanical integrity to withstand the reshaping forces exerted by the lenses (4).

Although extensive research has investigated CCT in adults, studies focusing on pediatric populations remain limited and show inconsistent results. This study aims to assess age-related changes in CCT across different pediatric age groups and to explore the relationships between CCT and other ocular biometric parameters in children, including axial length (AL), corneal curvature (Km), anterior chamber depth (ACD), and lens thickness (LT), thereby providing valuable data to support early diagnosis, personalized myopia management, and the safe application of orthokeratology in pediatric ophthalmology.

## 2 Materials and methods

### 2.1 Study participants

This retrospective observational study included 393 children (786 eyes), aged 3 to 17 years, who attended the Department of Ophthalmology at Beijing Shijitan Hospital, Capital Medical University, between June and July 2023. All participants underwent comprehensive ophthalmic evaluations during routine outpatient visits.

Inclusion criteria were as follows: (1) complete ocular biometric data including CCT, AL, ACD, and Km; (2) best-corrected visual acuity (BCVA) of ≥20/40 for children aged 3–4 years and ≥20/20 for those aged ≥5 years; and (3) provision of written informed consent by a parent or legal guardian, along with verbal or written assent from the child, when appropriate.

Exclusion criteria were: (1) history of ocular trauma or intraocular surgery; (2) presence of ocular surface or intraocular diseases (e.g., conjunctivitis, keratitis, cataract, glaucoma, retinal disorders); (3) systemic conditions affecting ocular development (e.g., diabetes, Marfan syndrome, Down syndrome); (4) strabismus or amblyopia; (5) current or prior use of contact lenses, orthokeratology, atropine, or other myopia control therapies; (6) poor cooperation due to impaired fixation, nystagmus, or neurological deficits.

The study was conducted in accordance with the Declaration of Helsinki and approved by the Ethics Committee of Beijing Shijitan Hospital, Capital Medical University (IIT2025-082-001). Informed consent was waived by our institutional because this is a retrospective observational study.

### 2.2 Data collection

Baseline demographic and clinical data were collected from medical records and included age, sex, height, weight, medical history, and medication use.

Ocular biometric measurements were performed using the ZEISS IOLMaster 700 (Carl Zeiss Meditec AG, Jena, Germany) by a single experienced technician. Parameters measured included CCT, AL, Km, ACD, and LT, all under natural pupil conditions. For myopic participants, cycloplegic refraction was obtained using compound tropicamide (Xingqi Pharmaceutical, Shenyang, China), administered bilaterally every 5 min for a total of six doses, followed by 30 min of occlusion. Refraction was measured via retinoscopy, and the spherical equivalent (SE) was calculated as SE = S + (C/2), where S denotes the spherical power and C the cylindrical power. Myopia was defined as a SE of ≤ − 0.50 diopters (D).

In this study, data from both eyes were included in the analysis to retain information from participants with unilateral myopia. Among the participants, 158 children underwent repeat ocular biometric measurements using the same ZEISS IOL Master 700 device 1 year after baseline to evaluate longitudinal changes.

### 2.3 Statistical analysis

Data were analyzed using SPSS version 21.0 (IBM Corp., Armonk, NY, USA). The normality of continuous variables was assessed using the Shapiro–Wilk test. Independent samples t-tests were used to compare CCT between sex and myopic and non-myopic groups. Spearman correlation analysis was employed to examine associations between CCT and age, AL, Km, ACD, LT, and SE. Paired samples t-tests were used to evaluate longitudinal changes in CCT over 1 year. Statistical significance was set at *p* < 0.05.

Sample Size Estimation: Based on preliminary data from our research group, a correlation coefficient of *r* = 0.15 was assumed for sample size estimation. Using the sample size formula for correlation analysis based on Fisher’s z transformation, with a significance level of *α* = 0.05 and a statistical power of 1 − *β* = 0.80, the minimum required sample size was calculated to be 346 participants. In this study, the actual sample size exceeded the required number, indicating sufficient statistical power to detect weak correlations.

## 3 Results

### 3.1 General characteristics

A total of 393 children were included, comprising 192 males (48.84%) and 201 females (51.16%), with a mean age of 9.5 ± 3.1 years. The age distribution was as follows: 3–6 years (18.06%), 7–9 years (32.32%), 10–12 years (31.30%), and 13–17 years (18.32%). Bilateral myopia was observed in 191 children (48.6%), unilateral myopia in 17 (4.33%), and no myopia in 185 (47.07%). CCT values ranged from 464 μm to 653 μm, with a mean of 551.53 ± 32.93 μm. Details are presented in Table 1.

**Table 1:** Distribution by gender, age, and myopia status.

Characteristic	Category	Number (%)
Gender	Male	192 (48.84%)
	Female	201 (51.16%)
Age (years)	3–6	71 (18.06%)
	7–9	127 (32.32%)
	10–12	123 (31.30%)
	13–17	72 (18.32%)
Myopia status	Bilateral	191 (48.6%)
	Unilateral	17 (4.33%)
	Non-myopic	185 (47.07%)

### 3.2 Association between sex, age, and CCT

**Figure 1:**
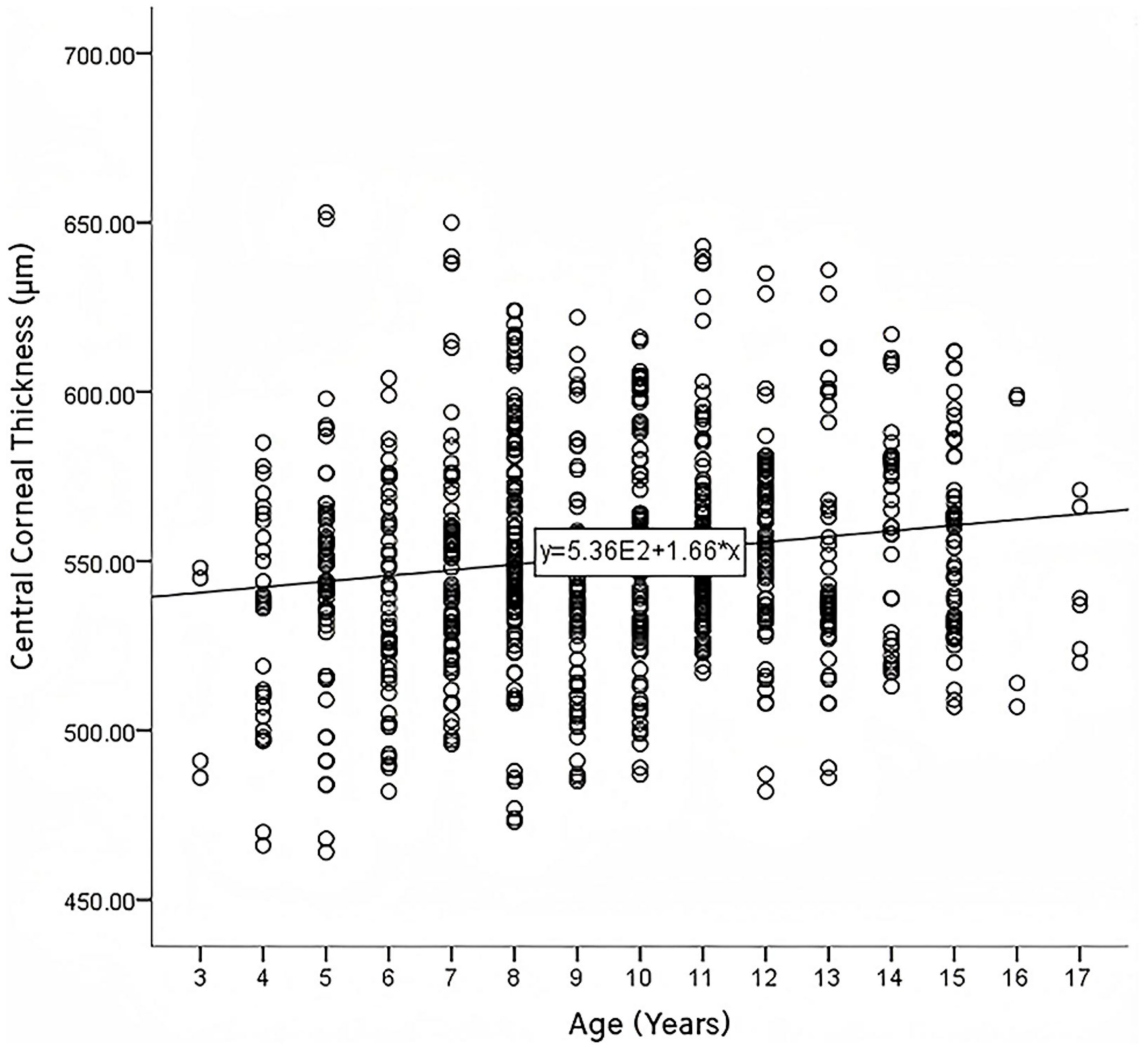
Scatter plot of the correlation between age and central corneal thickness.

No significant difference in CCT was observed between males and females (*p* = 0.142), with mean values of 553.29 ± 30.59 μm in males and 549.84 ± 34.98 μm in females. A weak positive correlation was observed between CCT and age (*r* = 0.150, *p* < 0.001), as illustrated in Figure 1. However, subgroup analysis by age groups (3–6, 7–9, 10–12, and 13–17 years) did not reveal significant correlations within groups (see Table 2 for details).

**Table 2:** Correlation between central corneal thickness and age.

Age group	CCT (μm)	*r*	*p* value
All ages	551.53 ± 32.93	0.150	<0.001
3–6	539.03 ± 33.46	0.060	0.481
7–9	550.56 ± 34.13	−0.064	0.308
10–12	557.63 ± 30.28	−0.009	0.894
13–17	555.11 ± 31.39	0.046	0.583

Spearman’s rank correlation analysis was performed.

### 3.3 Correlations between CCT and other ocular biometric parameters

CCT showed a weak positive correlation with AL (*r* = 0.121, *p* = 0.001), a negative correlation with Km (*r* = −0.239, *p* < 0.001), a very weak positive correlation with ACD (*r* = 0.079, *p* = 0.026), and no significant correlation with LT (*r* = −0.046, *p* = 0.195). Details are presented in Table 3.

**Table 3:** Correlation between central corneal thickness and biometric parameters.

Parameter	*r*	*p* value
Axial length	0.121	0.001
Corneal curvature	−0.239	<0.001
Anterior chamber depth	0.079	0.026
Lens thickness	−0.046	0.195

Spearman’s rank correlation analysis was performed.

### 3.4 Impact of myopia on CCT and its relationship with refractive status

CCT was significantly thinner in myopic eyes than in non-myopic eyes, with mean values of 548.77 ± 34.37 μm in the myopic group (*n* = 399) and 554.20 ± 31.29 μm in the non-myopic group (*n* = 387) (*p* = 0.021). Furthermore, among myopic eyes, a weak but statistically significant negative correlation was found between SE and CCT (*r* = −0.163, *p* = 0.003), indicating that greater myopic refractive errors were associated with slightly thinner corneas.

### 3.5 Longitudinal changes in CCT over 1 year across pediatric age groups

Among the 158 children who underwent repeat biometric evaluation after 1 year, the mean CCT increased significantly from 555.53 ± 35.01 μm at baseline to 559.75 ± 36.28 μm (*p* < 0.001). Stratified analysis demonstrated significant increases in CCT within the 3–6, 7–9, and 10–12 year age groups (*p* < 0.05), whereas no significant change was observed in the 13–17 year age group (*p* > 0.05). Details are presented in Table 4.

**Table 4:** One-year changes in central corneal (CCT) thickness by age group.

Group	Baseline CCT (μm)	CCT after one year (μm)	*p* value
All ages	555.53 ± 35.01	559.75 ± 36.28	<0.001
3–6	539.1 ± 23.86	545.4 ± 24.92	<0.001
7–9	555.4 ± 37.45	560.6 ± 37.62	<0.001
10–12	558.07 ± 33.15	561.61 ± 35.33	<0.001
13–17	567.3 ± 37.79	568.2 ± 42.07	0.492

Paired samples *t*-test was used for comparisons.

## 4 Discussion

While A-scan ultrasound pachymetry remains a traditional gold standard for measuring CCT (5, 6), its accuracy is highly operator-dependent, requiring precise centration and perpendicular probe alignment with the corneal surface (7). Additionally, topical anesthesia can induce corneal epithelial edema and disrupt the tear film, often causing CCT overestimation (8). In contrast, the IOL Master 700 used in our study is a non-contact swept-source OCT-based biometer that delivers rapid and accurate measurements along the visual axis. It has shown strong concordance with ultrasound pachymetry in assessing CCT (9) and allows simultaneous evaluation of multiple ocular parameters, including axial length, corneal curvature, anterior chamber depth, and lens thickness. This improves measurement efficiency, reduces cross-infection risk, and enhances patient comfort. Using this device, we assessed CCT in a large pediatric cohort aged 3 to 17 years and observed an age-related increase in CCT, particularly in younger children. CCT was significantly thinner in myopic eyes compared to non-myopic eyes and showed a weak but significant negative correlation with myopic refractive error and average corneal curvature. No significant associations were found between CCT and gender, axial length, anterior chamber depth, or lens thickness. We also analyzed longitudinal changes in CCT over 1 year and its relationship with lens thickness in children.

The relationship between CCT and gender remains inconclusive in the literature. Some studies suggest that males tend to have thicker corneas than females, a finding consistent across different racial and ethnic groups (10). Similar findings have been reported in pediatric populations (11, 12). However, other studies found no significant association between gender and CCT in various regions (13–15). For instance, Jing et al. reported no significant gender differences in corneal thickness among children (16). Consistent with their findings, our study also found no significant association between CCT and gender.

There is conflicting evidence regarding the relationship between CCT and age. Mimouni et al. and Hoffmann et al. reported no significant association between CCT and age (17, 18), whereas Hussein et al. observed that CCT increases from birth and reaches adult levels by 5–9 years (19). Similarly, the Pediatric Eye Disease Investigator Group reported an age-related increase in CCT between 1 and 11 years, followed by a plateau thereafter, with more pronounced changes in younger children (20). In our study, a weak positive correlation was observed between CCT and age; however, no significant associations were found within individual age subgroups (3–6, 7–9, 10–12, and 13–17 years), likely due to limited age variability within subgroups. In contrast, longitudinal analysis demonstrated significant increases in CCT in children aged 3–12 years, with no significant change after 13 years, consistent with a developmental plateau. These findings align with previous evidence suggesting that CCT increases during childhood and stabilizes in early adolescence. Importantly, narrow age ranges within cross-sectional subgroups and large individual variations may mask the age-related trend, whereas longitudinal data can more reliably reflect the real developmental changes in CCT.

Studies on the relationship between CCT and axial length have also yielded inconsistent results. Some reported no significant correlation between the two (21–23), while others, such as Peng et al., found a negative correlation between CCT and axial elongation rate, suggesting that thinner CCT may be associated with faster myopia progression (24). Wang et al. observed thicker corneas in eyes with longer axial length (25). In our study, a weak positive correlation was observed between CCT and axial length (*r* < 0.3), suggesting limited clinical significance. One hypothesis is that axial elongation primarily affects the posterior segment and sclera, leaving the cornea largely unaffected (26). Pedersen et al. modeled corneal thickness changes due to axial elongation and found that actual CCT change was only one-fourth of the theoretical value, further indicating that axial elongation may not significantly impact corneal thickness (27).

Large-scale clinical studies have explored the relationship between corneal curvature and CCT, though findings have varied. Nangia et al., in a study of 4,711 participants, found that increased corneal curvature was associated with thinner CCT (28). Similarly, Su et al. reported a significant negative correlation between CCT and corneal curvature (K value) in 3,280 Malay individuals in Singapore (29). However, other studies such as those by Shimmyo et al. (U.S.) and Sawada et al. (Japan) reported a positive correlation (10, 30), while some found no correlation (31). In our study, we observed a negative correlation between average corneal curvature and CCT, aligning with the findings of Nangia and Su.

Studies examining the relationship between CCT and ACD or LT are limited. In our study, the correlation coefficient between CCT and ACD was <0.1, indicating a very weak and clinically insignificant relationship. This is consistent with findings by Chen et al. and Jing et al. (16, 31). No significant correlation was observed between CCT and LT in our cohort. Jonas et al., in a study of 9,046 eyes from 4,610 adults, reported a positive correlation between CCT and LT (32). However, their study population had a mean age of 49 years, whereas our study involved children. Age-related lens thickening and other factors may affect the CCT–LT relationship, and thus the applicability of those adult findings to pediatric populations remains uncertain. Overall, data on the relationship between CCT and LT in children are scarce and warrant further investigation.

The association between CCT and myopia also remains controversial. Chang et al. reported that myopic eyes had thinner corneas in a cohort of 216 young adults with a mean age of 22.2 years (33). In contrast, Fam et al. found no association between CCT and degree of myopia in a study of 714 Singaporean Chinese individuals (34). In our study, CCT was significantly thinner in myopic compared to non-myopic eyes. Within the myopic group, a weak negative correlation was found between refractive error and CCT, indicating that greater myopic refractive error is associated with thinner corneas. These results are in line with those of Suzuki et al., who found a negative correlation between CCT and refractive error in 7,313 Japanese individuals (35). The Pediatric Eye Disease Investigator Group also found that for every 1D shift toward myopia, CCT decreased by an average of 1 μm in children (20).

A few limitations of this study should be acknowledged. First, both eyes were included in the analysis to preserve clinical information, which may introduce inter-eye correlation and should be considered when interpreting the results. Second, the 1-year follow-up cohort was relatively small due to the retrospective nature of the study, as only children with available follow-up data during routine clinical visits were included. Finally, the study was conducted in a single center, which may limit the generalizability of the findings to other populations.

## 5 Conclusion

In summary, this study evaluated CCT and its associations with ocular biometric parameters in children aged 3–17 years, based on cross-sectional and one-year longitudinal data obtained using the IOL Master 700. The results demonstrate that CCT increases with age, particularly in younger children, and tends to stabilize between 9 and 12 years. A negative correlation was observed between CCT and both average corneal curvature and myopic refractive error. No significant associations were found with gender, axial length, anterior chamber depth, or lens thickness. These findings provide updated insights into CCT development during childhood.

## Data Availability

The original contributions presented in the study are included in the article/supplementary material, further inquiries can be directed to the corresponding author.
